# Resources recovery from domestic wastewater by a combined process: anaerobic digestion and membrane photobioreactor

**DOI:** 10.1007/s11356-024-34468-3

**Published:** 2024-07-30

**Authors:** Elvira Ferrera, Ignacio Ruigómez, Carolina Vela-Bastos, Alice Ferreira, Luisa Gouveia, Luisa Vera

**Affiliations:** 1 Departamento de Ingeniería Química y Tecnología Farmacéutica, Facultad de Ciencias, Universidad de La Laguna, Avenida Astrofísico Francisco Sánchez S/N, 38206 La Laguna, Spain; 2 LNEG - UBB - National Laboratory of Energy and Geology I.P., Bioenergy and Biorefineries Unit, Estrada Do Paço Do Lumiar 22, 1649-038 Lisbon, Portugal; 3 GreenCoLab - Green Ocean Technologies and Products Collaborative Laboratory, CCMAR, Algarve University, Faro, Portugal

**Keywords:** Microalgal-bacteria consortium, Biomethane, Domestic wastewater, Biostimulant, Circular economy

## Abstract

Anaerobic and membrane technologies are a promising combination to decrease the energy consumption associated with wastewater treatment, allowing the recovery of resources: organic matter as biomethane, nutrient assimilation by microalgae and reclaimed water. In this study, domestic wastewater was treated using a combination of an upflow anaerobic sludge blanket sludge reactor (UASB) and a membrane photobioreactor (MPBR). The outdoor facilities were operated continuously for three months under unfavourable environmental conditions such as lack of temperature control, winter season with lower solar irradiation and lower daylight hours which was a challenge for the present work, not previously described. The energetic valorisation of the organic matter present in the wastewater by biomethane produced in the UASB would contribute to reducing overall facilities’ energy requirements. The ultrafiltration (UF) membrane facilitated the harvesting of biomass, operating at 10 L·h^−1^·m^−2^ during the experimental period. Although the main contribution to fouling was irreversible, chemical cleanings were not necessary due to effective fouling control, which prevented the final TMP from exceeding 25 kPa. In addition, microalgae-bacterial consortium developed without prior inoculation were harvested from the MPBR using membrane assistance. The obtained biomass was also successfully tested as a biostimulant for corn germination/growth, as well as a biopesticide against *Rhizoctonia solani* and *Fusarium oxysporum*.

**Graphical abstract:**

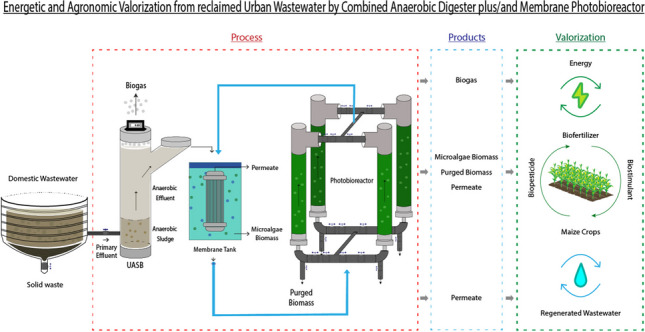

**Supplementary Information:**

The online version contains supplementary material available at 10.1007/s11356-024-34468-3.

## Introduction

According to the Food and Agriculture Organisation (FAO 2023), water scarcity hits more than 40% of people worldwide. Moreover, over 5 billion may face drinking water shortage by 2050 (3.6 billion in 2018), due to an inadequate global water source management (mainly in Asian and African countries). Crops irrigation is a critical factor in the integrated water cycle management that allows the production of 40% of the world’s food while utilising only 20% of the total cultivated land (FAO 2017). The traditional relationship between agriculture and water needs to be reformulated to face up the challenges of the climatic change and growing population.

One of the most cultivated crops in the world is maize or corn, whose production reached 1.2 billion tons in 2021 occupying around 205 million hectares (FAO 2021). In Tenerife (Canary Islands, Spain), 39% of the agricultural surface dedicated to cereals crop is for corn cultivation and more than 50% of this is under irrigation to increase final production (Gobierno de Canarias n.d.). Tenerife, like other isolated areas with limited natural water resources, suffers water stress, having opted for seawater desalination, but especially to reuse reclaimed domestic wastewater to meet agricultural irrigation needs. The use of eco-friendly technologies and solutions for the management and treatment of these non-conventional resources, aligned with the circular economy and sustainable development strategy, are necessary to face up the climate emergency and the growing water demand by the agricultural sector.

The use of anaerobic systems combined with membrane photobioreactors (MPBRs) is probably one of the most promising options for the future of domestic wastewater treatment. Anaerobic digestion permits lower sludge production compared to aerobic conventional technologies and allows energy recovery through biogas production (Cecconet et al. 2022). Although the technology is widely accepted, temperature and low organic concentration can limit biogas generation. Nevertheless, several laboratory studies have achieved favourable energy production at low values of chemical oxygen demand (COD) and psychrophilic conditions (Serrano-León et al. 2018; McCarty et al. 2011). In this way, the upflow anaerobic sludge blanket (UASB) reactor is receiving renewed attention (Vassalle et al. 2022; Ferrera et al. 2022; Mainardis et al. 2020; Ribera-Pi et al. 2020) and could be suitable for the Canary Islands, where a subtropical oceanic climate predominates, with all year-round moderate temperatures due to the proximity to the sea and the trade winds.

Nevertheless, the effluent from UASB reactors still contain high concentrations of nitrogen and phosphorus which limit their secure discharge in the water bodies (European Commission 1998). On the contrary, microalgae can capture these water pollutants as nutrients for their growth and promote the advanced treatment of secondary wastewaters (WW), while reducing microalgae production costs (Michalak and Chojnacka 2016). The membrane photobioreactor (MPBR) allows microalgae cultivation in the photobioreactors (PBRs) and micro or ultrafiltration membrane facilitates microalgae harvesting (Luo et al. 2018). This technology reaches biomass concentration 3.5 times higher than conventional PBRs and distinguishes hydraulic retention time (HRT) and solid retention time (SRT), achieving significant nutrient removal (Gao et al. 2018; Kumar et al. 2010).

On the other hand, population growth and attempts to increase agricultural production have led to an excessive use of synthetic fertilisers worldwide. Synthetic fertilisers are known to contribute to greenhouse gas emissions and in situ soil degradation which can lead to infertility and biodiversity losses, and human health risks directly related to their use. Moreover, their misapplication can also lead to water pollution events such as eutrophication and groundwater contamination (FAO 2021). Therefore, the search for organic fertilisers for promoting the development of healthy plants and crops and soil fertility and quality is being extensively studied. Among these, those resulting from microalgae have generated great interest for their application to sustainable agriculture (Ferreira et al. 2023; Ferreira et al. 2021; Viegas 2021a, b, c). This has been due to their content of bioactive compounds such as phytohormones, amino acids or polysaccharides (Ranglová et al. 2021), and their ability to improve nutrition assimilation of crops and expand their resistance to biotic and abiotic stresses (Chiaiese et al. 2018; Ronga et al. 2019).

This work focused on the evaluation of a novel configuration for domestic wastewater treatment (WWT) at pilot scale: a secondary treatment carried out by an UASB unit and a tertiary treatment by a MPBR. This new system was also tested for resource recovery from primary wastewater. The wastewater reclamation efficiency, nutrients removal rate, microalgae biomass productivity and biogas production and composition were periodically monitored. The biogas production by the UASB was evaluated and the agronomic potential of the resulting fractions (microalgae, waste biomass and permeate), was assessed. The obtained microalgae biomass, without any pre-treatment, was evaluated as a biostimulant for corn germination and growth, as well as the biopesticide effect against *Fusarium oxisporum* and *Rhizoctonia solani* fungi.

The study targets to comply with Sustainable Development Goals, namely SDG2 Zero Hunger, SDG6 Clean Water and Sanitation, SDG7 Affordable and Clean Energy and SDG13 Climate Action).

## Material and methods

### Experimental setup and performance

**Fig. 1:**
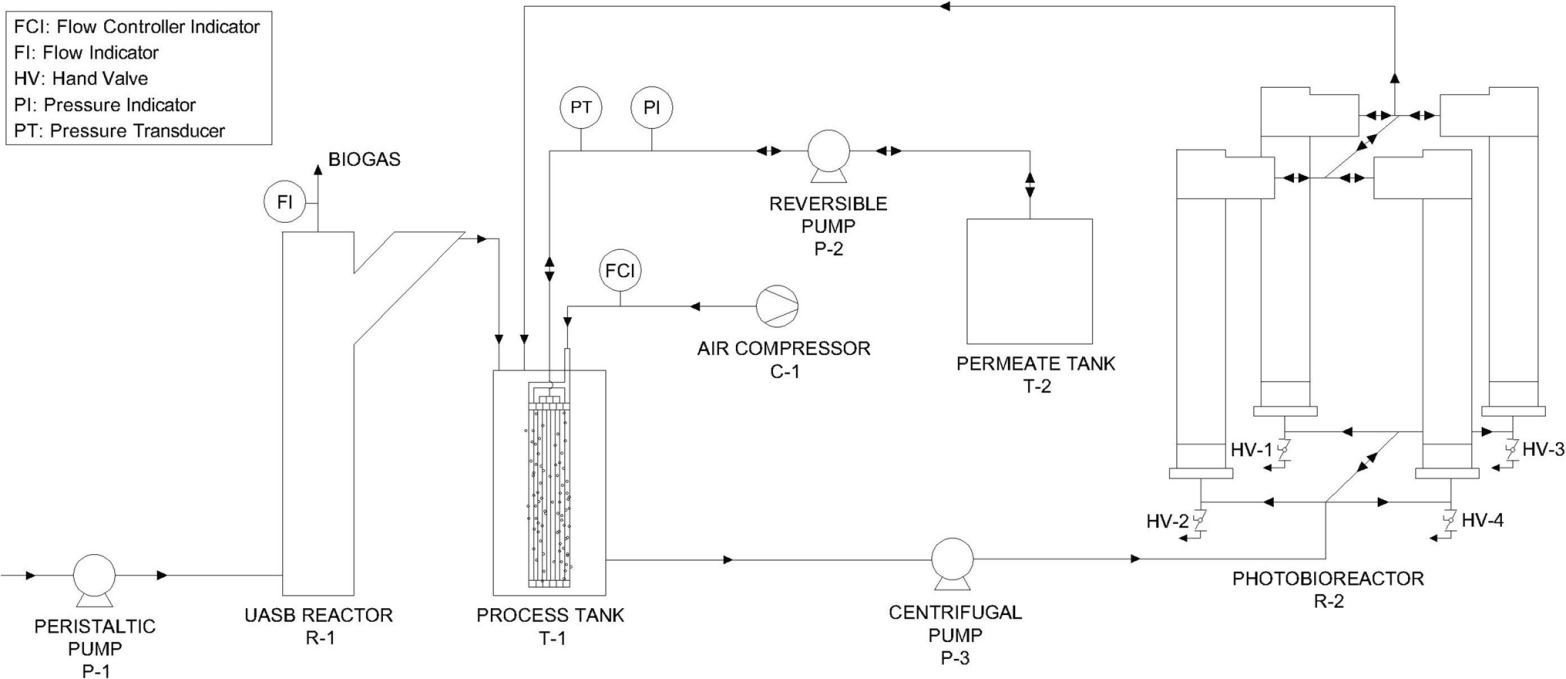
Flow diagram of the UASB-MPBR pilot unit

A representative fraction of real domestic wastewater collected by the Tenerife Northeast Wastewater Treatment Plant-WWTP (Canary Islands, Spain) was treated by a technological train composed of an anaerobic digester (UASB) followed by a membrane photobioreactor (MPBR) (Fig. 1). The main characteristics of the feedwater are presented in Table 1. It is important to emphasise that the variability observed in the data is primarily due to occasional discharges into the sewer network from agricultural and industrial activities (mainly slaughterhouse and soft drinks industries) taking place in the vicinity of the WWTP, as noted in previous studies (Ruigómez et al. 2016).

**Table 1:** Characterisation of feedwater to system pilot (UASB + MPBR). Values are given as mean ± standard deviation

Parameter	Units	Mean ± sd
pH	-	7.6 ± 0.3
EC	μS·cm^−1^	1813 ± 68.3
Turbidity	NTU	206.8 ± 35.7
TSS	mg·L^−1^	152.5 ± 25.8
VSS	mg·L^−1^	151.3 ± 22.5
HCO_3_^−^	mg·L^−1^	154.8 ± 11.5
COD	mg·L^−1^	545.4 ± 71.1
COD_s_	mg·L^−1^	249.7 ± 53
DOC	mg·L^−1^	79.4 ± 22.8
N-NH_4_^+^	mg·L^−1^	67.4 ± 9
TP	mg·L^−1^	12.5 ± 1.4
SO_4_^2−^	mg·L^−1^	48.5 ± 1.8

#### Upflow anaerobic sludge blanket (UASB) pilot unit

The UASB reactor acted as secondary treatment of domestic wastewater which was previously subjected to degreasing and screening. It was inoculated with 24 L of anaerobic sludge from a sludge digester from a conventional domestic WWTP and stabilised for 6 months. The reactor was continuously operated under psychrophilic conditions at an average temperature of 20 °C. The UASB pilot unit has a total volume of 115 L, equipped with four valves at different heights (0.1, 0.4, 0.7 and 1.0 m) for sampling, while the effluent leaves the reactor at a height of 1.7 m. Additionally, the experimental setup was located outdoors without any thermal isolation. During the experimental period, the solar irradiation (daily average irradiance 2717–3964 W·h·m^−2^) and temperature (12.5–25 °C) were the lowest recorded values in the year. Feedwater was continuously pumped to the UASB unit by a peristaltic pump (520SN Watson Marlow, Falmouth, UK) at an organic loading rate (OLR) of 2.39 kg COD·m^−3^·d^−1^. The effluent flowrate was 21 L·h^−1^, involving an upflow rate of 0.3 m·h^−1^ and HRT of 5.5 h with periodical purges of biomass (0.6 L once per week for biomass analysis).

Regarding to biogas production and methane productivity, biogas flowrate was measured with a gas-metre (Milligas Counter MGC-1 PMMA, Ritter Apparatebau GmbH & CO. KG, Germany) and its relative composition was determined using gas chromatography with He as carrier gas at a flowrate of 36 mL·min^−1^ (7820A GC Agilent, Santa Clara, United States). Subsequently, the biogas yields of the raw materials, calculated and corrected to standard volumes, have been referred to the removed dissolved COD (APHA 2005).

#### Pilot membrane photobioreactor (MPBR)

The MPBR consisted of a photobioreactor with 4 vertical tubular modules made up by polymethyl methacrylate and a volume of 47 L each. The PBR was connected in series to a membrane tank (MT). Microalgae cultivation was carried out into the upflow tubular closed PBR and the membrane separated the microalgae biomass from the water effluent (permeate), avoiding its wash-out. The 180 L MT was equipped with an immersed ultrafiltration module ZeeWeed ZW-10 (SUEZ-Water Technologies and Solutions, Canada) composed by hollow polyvinylidene fluoride (PVDF) fibres with an external diameter of 1.9 mm, a mean pore size of 0.04 μm and a total filter surface of 0.93 m^2^. The permeate was produced by a slight vacuum at a constant permeate flux, with a double-direction micro-gear magnetic pump, which held both processes, membrane filtration and backwashing (Micropump-GA, Idex Corporation, Vancouver, WA USA). The transmembrane pressure was monitored with a pressure transducer (BD SENSORS, Germany) to assess membrane fouling. During the experimental period, a constant permeate flux of 10 L·h^−1^·m^−2^ was applied for 450 s, and a backwash flux of 30 L·h^−1^·m^−2^ was applied during 30 s between filtration cycles. Air flow at 5 NL·min^−1^ was supplied with a compressor (Secoh–Shanghai Mec, Japan – China) at the bottom of the membrane tank to avoid biomass sedimentation and assure homogenisation. In addition, a peristaltic pump (Easy-Load Masterflex, Cole-Parmer, USA) was used to keep the HRT constant. All sensors and operational parameters were monitored and controlled using DAQ Factory software (AzeoTech® Inc., Ashland, OR, USA). Unlike the PBR, the MT was located indoors together with the monitoring equipment. The MPBR started its operation without any inoculum and the biomass development was carried out from the indigenous microorganisms present in the feedwater. Biomass was continuously homogenised in MPBR by a centrifugal pumping from the MT to the PBRs and it was periodically purged so that HRT and SRT were 1.5 d and 5 d, respectively.

### Maize development

#### Germination

The germination-inducing capacity of the microalgae consortium grown on MPBR, permeate from the membrane, and purge was evaluated for maize seeds (*Zea mays*). Four maize seeds were placed on Whatman filter paper in a Petri dish, in triplicate for each condition. A volume of 10 mL of microalgae consortium and purge is taken at concentrations at 0.2, 0.5 and 1 g·L^−1^, but also permeate after membrane (directly). Distilled water was used as the negative control. Then, the seeds were incubated at 20 °C (FITOCLIMAS600, Aralab, Portugal) for 8 days. After the incubation period, the seedlings were carefully separated, and the roots were measured using ImageJ software.

The germination index (GI) of each sample was determined by the Eq. 1 (Zucconi et al*.,* 1981):

1 $$GI \left(\%\right)=\left(\frac{G\times L}{{G}_{w}\times {L}_{w}}\right)\times 100$$

where *G* and *L* are the number of germinated seeds and the root length for each condition and *G*_*w*_ and *L*_*w*_ are the same parameters for the control (distilled water). Results higher than 100 show a positive effect on germination.

#### Plant growth

**Fig. 2:**
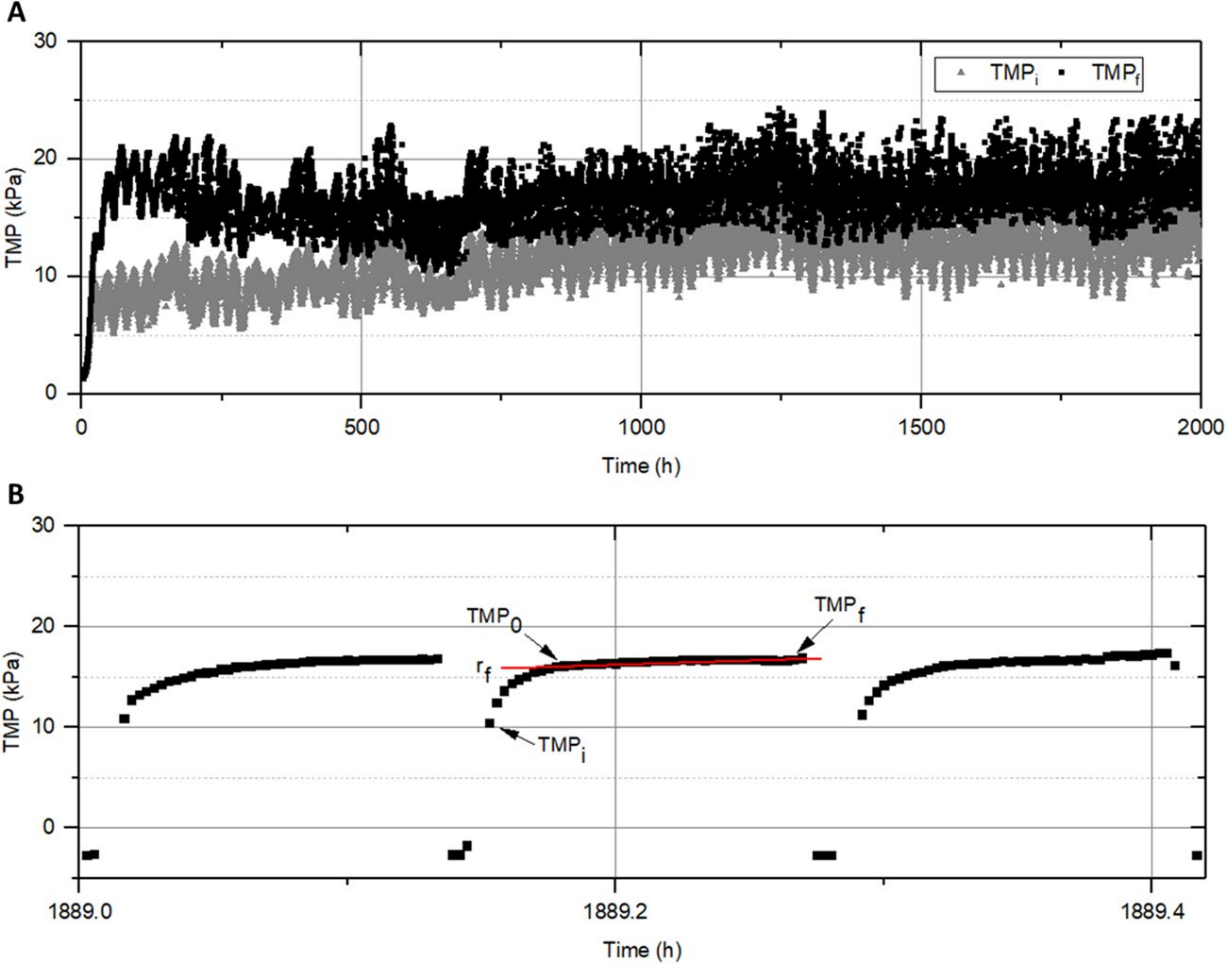
**A** Evolution of transmembrane pressures at the beginning (TMP_i_) and the final (TMP_f_) of each filtration cycle. **B** Transmembrane pressure evolution with elapsed time for consecutive filtration/backwashing cycles

The maize seedlings were transplanted into 1.2-L pots containing a mixture of soil and perlite at a ratio of 50:1 (Fig. 2). Each condition was made in quadruplicate. The same conditions used for the germination trials were applied: microalgae consortium (0.2, 0.5 and 1 g·L^−1^), purge (0.2, 0.5 and 1 g·L^−1^) and permeate. Additionally, a positive control using synthetic nutrients, known as Hoagland solution, was included.

The growth progress of the maize plants was measured every 20 days, from the beginning of the stem to the top of the plant. At the end of the growth test (64 days), the maize plants were carefully separated in roots and stem with leaves and dried in an oven at 105 °C to measure their dry weight.

#### Biopesticide bioassays

The fungi *Fusarium oxysporum* sp. *melonis* and *Rhizoctonia solani* were used for the biopesticide bioassays. Agar 2% and potato dextrose agar (PDA) (4 mg·L^−1^ of potato starch, 20 mg L^−1^ of dextrose, 15 mg L^−1^ of agar) were prepared to carry out the assays. Tartaric acid (10% w/v) at 1% (w/v) was added to PDA to decrease the pH of the medium to 3.5 and inhibit bacterial growth. Regarding the procedure, sterile Petri dishes were used, where 4 Oxford towers were placed, to make the holes where the study sample were latter added. Agar was poured up to half of the dish and left to solidify. Then, PDA was added to fulfil the upper half of the Petri dish. Suspensions of the microalgae-bacteria consortium were added at concentrations of 5, 10 and 15 g·L^−1^ and the fungus was placed in the middle of the dish. In addition, sterile distilled water was used as the negative control and a commercial Rovral solution at 10 mg·L^−1^ was used as the positive control. Afterwards, the dishes were incubated in the dark at 25 °C for 7 days for *F. oxysporum* and 3 days for *R. solani*, according to Srivastava et al. (2011) and Mayo et al. (2015), respectively. Subsequently, the inhibition percentage was calculated using Eq. (2).

2 $$I \left(\%\right)=100-\left(\frac{PD}{CD} \times 100\right)$$

where *I* correspond to the inhibition (in %), *PD* to the diameter of the fungus growth with the suspension of microalgae, purge and *CD* to the control (distilled water).

### Analytical methods

#### Domestic and reclaimed wastewaters characterisation

Water samples collected from inlet (feedwater) and outlet of UASB and permeate from the MPBR units. Chemical oxygen demand (COD), total solids (TS), volatile solid (VS), total suspended solids (TSS), volatile suspended solids (VSS), turbidity, pH and electrical conductivity (EC) were determined according to standard methods (APHA 2005). The concentrations of ammonium (NH_4_^+^), nitrites (NO_2_^−^) and nitrates (NO_3_^−^) were quantified by ion chromatography (Methrom 882, Herisau, Appenzell Ausserrhoden, Switzerland). Total phosphorus was measured with LCK350 kit (Hach Lange GmbH, Willstätterstraße, Germany) and the dissolved organic carbon (DOC) was measured with a TOC-metre (multi N/C 3100, Analytik Jena, Jena, Germany) in previously filtered samples (1.2 μm). Titration was employed to determine total alkalinity (TA) and partial alkalinity (PA). The difference between TA and PA is the intermediate alkalinity (IA), which is usually related to volatile fatty acids (VFA) (Ripley et al. 1986).

#### Thermogravimetric analysis for biomass characterization

The thermogravimetric analysis was conducted using a Mettler Toledo model TGA/DSC (EEUU) apparatus with high precision weighing capabilities (± 0.01%) and a sensitivity of 10 μg. The sample weight used for the experiment varied between 5 and 6 mg, depending on the biomass density. The measurements were conducted in a nitrogen flow of 0.05 L·min^−1^, with a heating rate of 283 K·min^−1^ with an uncertainty of ± 1 °C. The TG and DTG curves were analysed in the range of 473–773 K using a symmetric Gaussian curve (DTG_i_) model for each pseudo-component. Instead of model compounds, data from previous studies were used in accordance with Ferreira et al. (2020).

## Results and discussion

### UASB pilot unit performance and biogas production

The UASB pilot unit exhibited stable behaviour throughout the experimental period. The main characteristics of the UASB microbial suspension did not undergo significant changes, and anaerobic granular sludge was observed in the lower zone of the reactor (Figure S1). Table 2 shows the concentrations of TS, VS, COD and COD_s_ at different heights in the UASB reactor. The results revealed that the gradients of the parameters associated with granular sludge (TS, VS and COD) dramatically increased with depth showing the typical stratification of the sludge blankets. In contrast, the COD_s_ remained more stable throughout the reactor, and the effluent exhibited a significant lower value (32% of the inlet one) consistent with the reported TS retention (98%) in the UASB and the mentioned stratification phenomena. Additionally, the pH inside the reactor remained close to neutrality (7.6 ± 0.3), and the alkalinity ratio never exceeded 0.3 (IA/TA = 0.15 ± 0.01), indicating the absence of acidification due to VFA accumulation (Martín-González et al. 2013) Similarly to other studies on domestic wastewater treatment by anaerobic digestion, low nutrient removals were recorded. On the other hand, the reduction in COD and TSS concentrations achieved by the UASB was 55.6% (from 545 to 237 mg·L^−1^) and 44.4% (from 153 to 85 mg·L^−1^), respectively. Kim et al. (2011) investigated a two-stage anaerobic treatment system to treat synthetic wastewater. The first stage consisted of a fluidised anaerobic-bed bioreactor (AFBR), followed by post-treatment with an anaerobic fluidised-bed membrane bioreactor (AFMBR). The feed concentration to that combined system was 513 mg·L^−1^, like the values reported in the current study, achieving COD removal of 88% in the AFBR and 87% in the AFMBR. These high percentages of reclamation may be due to the experimental study was carried out at laboratory scale under controlled operating conditions (mesophilic temperature (35 °C) and low TSS concentrations (14 mg·L^−1^)). In fact, subsequent studies with real wastewater at pilot scale by the same research team (Shin et al. 2014), operating with HRT values between 4.6 and 6.8 h and temperatures between 8 and 30 °C, revealed that, although the overall COD removal percentage of the system was over 90%, this value was considerably reduced in the first stage (i.e. equivalent treatment stage of the UASB reactor that feeds the membrane bioreactor, MPBR or AFMBR), reaching percentages close to 45% in the summer season and 15% in the winter season (Shin et al. 2014). It should be noted that both percentages were lower than the values reported in the current work. Regarding soluble sulphate reduction, the UASB pilot reactor allowed for a similar removal percentage (52.8%) to that observed by Shin et al. (2014) during the winter season, decreasing from 48.5 ± 1.8 to 22.9 ± 4.2 mg SO_4_^2−^ L^−1^.

**Table 2:** Characterization of the UASB reactor at different heights

Parameter	Units	Height 0 1.7 m	Height 1 1.0 m	Height 2 0.7 m	Height 3 0.4 m	Height 4 0.1 m
TS	g·L^−1^	0.88 ± 0.03	29.89 ± 1.18	32.67 ± 3.17	52.04 ± 3.85	52.2 ± 3.71
VS	g·L^−1^	0.47 ± 0.12	22.64 ± 1.03	29.09 ± 0.86	29.09 ± 0.86	39.25 ± 2.94
COD	g·L^−1^	0.26 ± 0.07	29.24 ± 3.21	23.34 ± 1.78	23.34 ± 1.78	44.5 ± 2.62
COD_s_	g·L^−1^	0.12 ± 0.05	0.38 ± 0.03	0.36 ± 0.02	0.38 ± 0.01	0.3 ± 0.06

On the other hand, the production and composition of biogas generated in the UASB pilot unit remained stable despite fluctuations in the feedwater. The average methane fraction ranged between 71 and 79%. Additionally, the conversion of organic matter, expressed as the COD removal capacity, was 1.3 g COD·L^−1^·d^−1^, which is comparable to the values reported by Serrano-León et al. (2018) under similar HRT conditions. However, in that study, the authors observed a higher methane production than the present one (120 mL CH_4_·g COD_removed_^−1^ and 25 mL CH_4_·g COD_removed_^−1^, respectively). This disparity could be attributed to the differences in reactor scale (pilot vs. lab) and the lack of control on the environmental conditions in outdoor UASB (without thermal isolation). These findings are consistent with Lew et al. (2011), who highlighted the significant impact of temperature on COD removal efficiency. In fact, this work showed removal percentages of 72 and 68% at 20 °C and 14 °C, respectively. Additionally, Alvarino et al. (2016) obtained specific CH_4_ yields ranging from 0.013 to 0.016 L CH_4_·g TSS^−1^·d^−1^, operating outdoors with an UASB reactor of 120 L. This value was similar to that obtained in the present study (0.014 L CH_4_·g MLVSS^−1^·d^−1^) and to that reported by Ruigómez et al. (2016) (0.012 L CH_4_·g MLVSS^−1^·d^−1^) during the treatment of domestic wastewater (MWW) from the Tenerife Northeast WWTP using an anaerobic immersed membrane bioreactor (AnMBR). However, it is worth noting that the biogas flow rate (≈ 90 L·d^−1^) reported by Alvarino et al. (2016) was approximately double that obtained in our UASB. This difference could be attributed to the authors operating with a longer HRT (12 h) feeding synthetic water from skimmed milk with a high organic matter content and temperatures between 20 and 22 °C.

In addition, a key obstacle faced by anaerobic processes working at low temperatures (< 20 °C) is the reduction of the hydrolysis of particulate organic matter into soluble molecules, decreasing the efficiency of the overall process. In the current work, the removal percentage of particulate organic matter was 31 ± 10.3% which is slightly higher than the values reported by Ribera-Pi et al. (2020) using a granular AnMBR (23 ± 17%). In the same work, the authors also studied two more pilot scale reactors, a flocculent UASB and a conventional AnMBR. The three reactors were fed with low-strength municipal wastewater (a soluble COD of 54.1 ± 10.3 mg·L^−1^ and a particulate COD of 84.1 ± 48.5 mg·L^−1^, respectively) and operated at psychrophilic conditions (9.7 ± 2.4 °C). Ribera-Pi et al. (2020) reported higher hydrolysis percentages in flocculent reactors (54 ± 12 and 38 ± 17%, respectively) than in the granular AnMBR. However, no influence was observed on the methane production yield, which was higher in the anaerobic systems with membrane filtration, which could be due to an increase of the solid retention into the reactors.

Obviously, operating under psychrophilic conditions, along with the large temperature variability (12.5–25 °C) and the high content of particulate organic matter, could be related to lower metabolic activity of the microorganisms and reduced biogas production. However, these results are similar to those obtained by other authors who have worked with low-strength domestic wastewater using low HRT (Shin et al. 2014; Yoo et al. 2012).

### MPBR pilot unit

#### Wastewater treatment efficiency and microalgae biomass productivity

The MPBR pilot unit was fed with the anaerobic effluent of the UASB bioreactor. As expected, the post-treatment achieved complete removal of particulate matter, as the ultrafiltration membrane acted as a solid barrier. Furthermore, the MPBR significantly reduced the organic matter concentration in the permeate stream, with removal percentages for COD and D^OC^ of 68.5 ± 10.3% (from 237 to 73 mg·L^−1^) and 50.0 ± 19.7% (from 30 to 15 mg·L^−1^), respectively. These results suggest the existence of microalgae-bacteria consortium within the photobioreactor. Although microalgae can grow heterotrophically, nowadays, the removal of COD through different monocultures remains a challenge achieving rates of less than 13%, and lower than traditional activated sludge technology (Li et al. 2024). In contrast, the presence of heterotrophic microorganisms allows for the reduction of organic compounds, as recently demonstrated in a study conducted by Segredo-Morales et al. (2024). The authors achieved COD removal percentages like those obtained in the current MPBR, ranging from 48% (May–July) to 75% (July–October). That study was carried out using secondary effluent from a municipal WWTP with a vertical multicolumn upflow membrane photobioreactor, attributing the increase in COD reduction to a greater activity of heterotrophic bacteria.

Additionally, the pH stability of the MPBR influent, close to neutrality, prevented phenomena such as ammonium stripping or phosphate precipitation during the development of the microalgae-bacteria consortium. Therefore, the removal of nitrogen and phosphorus in the MPBR can be mainly attributed to the growth of biomass. The average biomass productivity (BP) of the system was 65.55 ± 8.55 mg VSS·L^−1^·d^−1^, where the typical range of values (60–200 mg VSS·L^−1^·d^−1^) has been reported in other MPBRs fed with effluents from anaerobic treatment processes of domestic wastewaters (González –Camejo et al. 2020, 2018). As previously stated, the anaerobic treatment’s inability to remove nutrients and transform nitrogen compounds led to incoming MPBR concentrations of total dissolved nitrogen ranging between 79.3 and 95.2 mg·L^−1^, identical to those reported for the feedwater. On average, the system was able to recover between 2.0 and 5.9 mg·L^−1^·d^−1^ of soluble total nitrogen under the limiting environmental conditions of illumination (2717–3964 Wh·m^−2^) and temperature (12.5–25.0 °C) recorded during the experimental study. The values reported by González-Camejo et al. (2018) were higher for a MPBR fed with anaerobic effluent under similar HRT and SRT (average nitrogen recovery rate of 15 mg N·L^−1^·d^−1^), but with additional continuous LED light offering a continuous light irradiance of 300 μmol·m^−2^·s^−1^ (light:dark cycle of 24:0 h) and slightly higher temperature (25 °C). It is worth noting that in the current study, the average measured value of dissolved inorganic nitrogen (DIN) was 72.4 ± 10.7 mg N·L^−1^ in the MT suspension but decreased to 62.6 ± 13.3 mg N·L^−1^ in the purge. This could be related to a flocculation process and a greater assimilation of nitrogen by the biomass accumulated in this stream. Neither nitrite nor nitrate were detected in the different samples collected in the MPBR, so the DIN decrease in the purged suspension appears to be associated with the assimilation of ammonium by the biomass.

Regarding phosphorus, the MPBR allowed for the removal of 22% of the total phosphorus present in the feedwater at a recovery rate of 4.8 to 6.0 mg P·L^−1^·d^−1^, slightly higher than the values reported by González-Camejo et al. (2018) (1.7 to 1.0 mg P·L^−1^·d^−1^).

Overall, the generated biomass allowed for the nutrient’s recovery from wastewater, although the values were lower than those observed in similar studies. Therefore, higher SRT should be considered for improving the nutrients recovery. Additionally, the phosphate content and the N:P ratio (8.7 ± 0.7 on average) observed in the permeate (higher than the N:P ratio necessary for the growth of microalgae) suggest that low light intensity and external shadows were the main limitations for the efficient MPBR performance. In fact, González-Camejo et al. (2020) and Ruiz-Martinez et al. (2012) reached the same conclusion using different types of closed MPBRs.

#### MPBR filterability

Figure 2 shows the evolution of the initial and final transmembrane pressures (TMP_i_ and TMP_f_, respectively) over the 3-month experimental period. TMP_i_ allows for the study of irreversible fouling by analysing the TMP at the beginning of the filtration cycles (i.e. right after the backwashing stage ends), while TMP_f_ quantifies the overall fouling of the membrane module at the end of the filtration phase.

As expected, at the beginning of the experiment, the difference between TMP_i_ and TMP_f_ was significant; indicating that fouling was mainly due to the external deposition of biomass (microalgae-bacteria consortium) on the surface of the module ZW-10. Therefore, during these initial cycles, the primary contribution to fouling was the external matter deposition (≈ 50%), suggesting that the attached foulants could be partially detached and re-dispersed in the bulk suspension by the action of the physical cleanings consisting of backwashing (J_B_ = 30 L·h^−1^ m^−2^; t_B_ = 30 s) aided by air scouring (5 NL·min^−1^). However, a significant contribution of irreversible fouling to the final transmembrane pressure (TMP_f_) achieved by the module was also observed, with a value of approximately 44% (the remaining 6% was due to the resistance that the membrane module offers to water passage). This early fouling consolidation has been observed in other MPBRs and is usually attributed to the presence of extracellular polymeric substances (EPS) and soluble microbial products (SMP) in the MT suspension, leading to the formation of a gel layer on the membrane surface as well as the presence of some pore-blocking foulants. In fact, a recent study identified gel layer formation as the main fouling mechanism during the investigation of different SRTs in a microalgal-bacterial membrane photobioreactor (MPBR). The authors suggested that environmental stress and fierce competition between microalgae and bacteria were the underlying reasons for the high production of EPS and SMP that fouled the membrane module (Zhang et al*.* 2022). This is consistent with the progressive increase in TMP_i_ up to 500 h of filtration, when the system reached a pseudo-steady state. Subsequently, the evolution of both pressures (TMP_i_ and TMP_f_) followed a parallel trend. At this point, the values of both parameters increased at rates of 4.8·10^−3^ kPa·h^−1^ and 4.7·10^−3^ kPa·h^−1^, respectively. This can be explained by the equilibrium reached between the foulants deposited on the membrane by convective forces and the material removed from the filtration module by dispersion forces.

Figure 2 B shows the TMP filtration profiles obtained in the pseudo-stationary state during the test. The results reveal two clearly differentiated zones based on the evolution of the TMP, which is consistent with previous studies (Vera et al. 2015). In the first zone, immediately after completing the backwashing cycle, there is a pronounced increase in transmembrane pressure from TMP_i_ to TMP_0_. This last one (TMP_o_) is a characteristic TMP value related to external residual fouling that depends on the nature of the foulants and the efficiency of the physical cleanings (Ruigómez et al. 2017). This behaviour is generally related to ineffective re-dispersion of the material detached during backwashing, leading to the rapid pre-deposition of the foulants that remain near the membrane at the beginning of the filtration phase. Indeed, previous studies have demonstrated how internal gel layers of the cake expand but do not effectively detach from the membrane surface (Ye et al. 2011). Moreover, this phenomenon is more pronounced when cake compaction is avoided by operating at permeate fluxes and transmembrane pressures moderate, as in the present study. Subsequently, the second zone of the curve, between TMP_0_ and TMP_f_, corresponds to a slow linear increase in TMP with the elapsed time, associated with a mechanism of reversible cake layer formation on the membrane surface, which can be described by the following expression:

3 $$TMP={TMP}_{0}+{r}_{f}\cdot t$$

Analysing the results based on the TMPs contributions in the pseudo-steady state, it is observed that the main contribution was irreversible fouling (74.6%), while residual external fouling accounts for 14.2%. On the other hand, the reversible fraction barely reached 5%, while the percentage due to the membrane module hardly varied (6.2%). These results indicate that although periodic chemical cleanings are necessary for the sustainable operation to recover values close to membrane permeability, optimization of operating conditions (filtration and physical cleanings) could improve MPBR process performance.

### Overall process performance

**Fig. 3:**
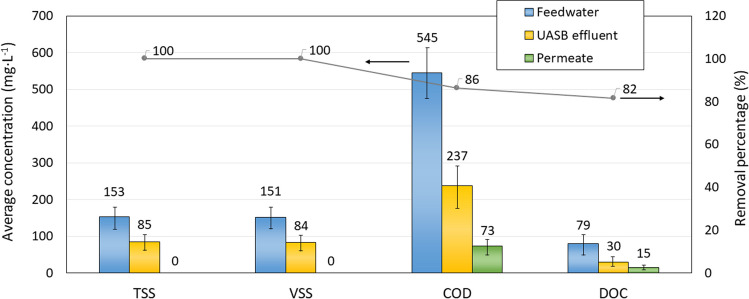
Evolution of average concentration of TSS, VSS, COD and DOC in the different streams of UASB + MPBR system: feedwater (inlet to UASB), UASB effluent and permeate from MPBR

Figure 3 shows the average removal efficiencies of the main parameters achieved with the overall system, combining UASB and MPBR technologies. Obviously, the membrane plays a relevant role regarding to the particulates retention for the global system and as it was foreseeable, acted as total barrier of this pollution indicator. The overall removal efficiency of COD and DOC was higher than 80% (86.4 ± 4.7% and 81.7 ± 5.1%, respectively). Despite the anaerobic treatment stage reduced the initial COD to moderate levels (approximately 237 mg·L^−1^), the MPBR post-treatment significantly improved the process performance, resulting in a particle-free effluent thanks to the ultrafiltration membranes. Additionally, the microalgae-bacteria consortium partially captured nutrients in the biomass, resulting in a permeate suitable for various uses, such as park irrigation, gardening, golf course maintenance, street cleaning or agriculture.

Regarding energy production, previous studies indicate that the available energy from methane combustion is 3.49 kWh·kg COD^−1^ (Shin et al. 2014; Yoo et al. 2012; Kimura et al. 2021). Therefore, the theoretical energy is 0.65 kWh·m^−3^, based on the organic fraction of COD recovered as CH_4_ (184.8 mg·L^−1^). Assuming a generator efficiency of 38% (Kimura et al. 2021), it can be estimated that the potential production of electrical energy will be 0.25 kWh·m^−3^. Although this production does not fully meet the energy demand of the UASB/MPBR system, it would significantly reduce operating costs, given that currently, under optimal conditions, the average specific energy requirements of large operational MBR plants can reach values of 0.4 kWh·m^−3^ (Krzeminski et al. 2017).

In summary, COD recoveries like those achieved in other studies where well-established technologies have been successfully implemented were obtained. In fact, submerged anaerobic membrane bioreactors (AnMBRs) commonly exceed COD removal percentages of 85% while operating at ambient temperatures (17.1 to 35 °C) (Shin and Bae 2018). Meanwhile, aerobic membrane bioreactors (MBRs) can achieve higher efficiencies, with COD removal rates usually ranging between 90–99% (Qrenawi and Rabah 2023). In the case of conventional activated sludge (CAS) processes, these efficiencies were reduced to 80–85% (Metcalf and Eddy 1995). In addition, it should be noted that the proposed system cannot fully replace the conventional technologies, but it can complement the wastewater treatment by promoting more sustainable practices and recover valuable resources present in domestic wastewater. This study has shown that even in unfavourable environmental conditions, the quality of the water treated by the UASB/MPBR system meets the physical–chemical and microbiological standards required for treated waters, especially owing to the incorporation of the membrane technology. In addition, the organic matter recovered and transformed into biogas/biomethane by the UASB could contribute to the self-consumption of energy of the UASB/MPBR system, which could even be more efficient if operating in co-digestion mode, incorporating the excess biomass produced in the MPBR into the UASB when agricultural demand decrease as was proved by Vassalle et al. (2022).

### Microalgae biomass valorization

#### Microalgae biomass as maize biostimulant

**Fig. 4:**
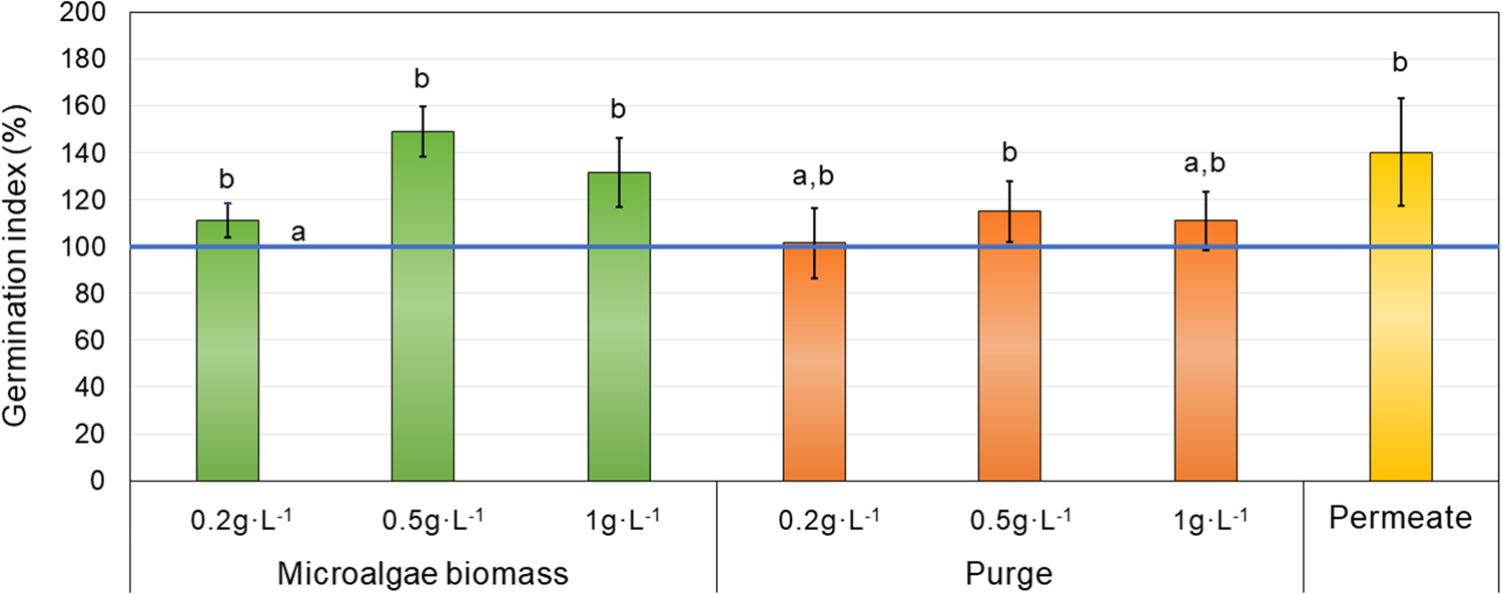
Germination index in maize seeds (%), considering distilled water as control (100%) for the biomass of microalgae in MPBR and MPBR purge at different concentrations (0.2, 0.5 and 1 g·L^−1^) and MPBR permeate. The error bars indicate the standard deviation (*n* = 12)

To quantify the germination of maize seeds, a *GI* of 100% has been considered for the control samples, when the seeds were treated with distilled water. Therefore, suspensions that gave *GI* values greater than 100% were considered to have biostimulant activity (Fig. 4 and Figure S2).

In general, it can be noted that all the evaluated biomass solutions (microalgae and purge), but also the permeate, have shown a positive effect on the germination index of maize seeds. It is worth noting that the results obtained at the concentration of 0.5 g·L^−1^ resulted to be the best one for both biomasses, showing a *GI* higher than 114.9% and 149% compared to the control in purge and microalgae mass, respectively. Nevertheless, the results highlight a greater efficiency for germination at all the tested concentrations for microalgae biomass. These results agree with the studies by Navarro-López et al. (2020) and Ferreira et al. (2021, 2023) where 0.5 g·L^−1^ was also the best concentration.

On the other hand, even though the microalgae biomass showed the higher germination index in this study, the *GI* reported by permeate is also noteworthy, achieving 140.2%. The dominant genus in the microalgae grown in the MPBR was *Chlorella* sp., which showed high GI values compared to previous studies conducted with biomass harvested in wastewater by Ranglová et al. (2021) and Morillas-España et al. (2022). These authors reported lower *GI* values in watercress seeds and even obtained negative effect on the *GI*, except in the case of 0.1 g·L^−1^ biomass concentration with a GI value of 3.5%, lower than the results obtained in this study. On the other hand, Amaya-Santos et al. (2022) also observed a higher *GI* in watercress seeds, reaching values of 9% using *Chlorella vulgaris* grown from urban wastewater.

Regarding other seeds, Viegas et al. (2021d) report positive values regarding germination with wheat at a concentration of 0.2 g·L^−1^ of *C. vulgaris* grown in poultry wastewater, with values that reach 147% germination. Also, Ferreira et al. (2021) determined *GI* for different seeds, using *C. vulgaris* grown in pig farm waters at a concentration of 0.5 g·L^−1^, concluding that the best germination rate was achieved in cucumber seeds, with a *GI* greater than 150%. To sum up, it has not been reported previous studies on *GI* for maize; therefore, it should be possible to obtain better *GI* values if different seeds are evaluated.

On the other hand, the thermogravimetry of the biomass suspensions revealed the percentage composition in terms of proteins, lipids and carbohydrates summarised in Table 3. These results seem to be aligned to the reported results by Rachidi et al. (2020). They demonstrated that polysaccharides are involved in the metabolic pathways of many plants and can act as biostimulants which allow to increase the crops quality and protect them against biotic and abiotics challenges.

**Table 3:** Composition (in percentage) of proteins, lipids, and carbohydrates of microalgal biomass and purge

	Composition (%)		
	Proteins	Lipids	Carbohydrates
Microalgae biomass	28.2	15.6	56.1
Purge	23.8	16.9	59.3

#### Microalgae biomass and purge as maize biofertiliser

**Fig. 5:**
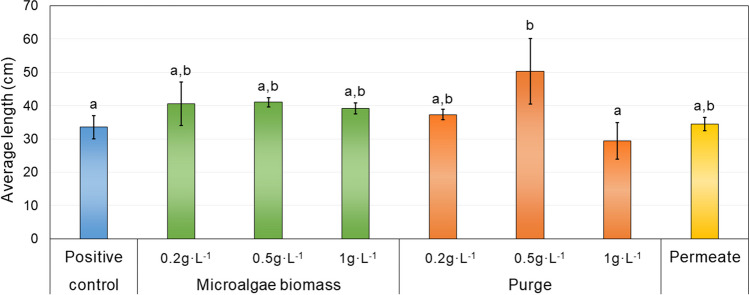
Length of the maize plant (cm), considering Hoagland as a control for the biomass of microalgae in MPBR and MPBR purge at different concentrations (0.2, 0.5, and 1 g·L^−1^) and MPBR permeate. The error bars indicate the standard deviation (*n* = 4)

The potential of both streams, microalgae biomass and purge, as biofertiliser for the development of maize plants was also studied (Fig. 5). It was carried out for 64 days, where the length of plants’ stems was measured at three different times. Although the results were similar for the several types of biomasses, the purge at 0.5 g·L^−1^ concentration showed the best results.

On the other hand, a similar general effect is observed in the roots and stems of the plants, both in size and weight. The lengths were equal to the positive control, except in the mentioned case of purge at 0.5 g·L^−1^, where positive differences are observed both, in dry weight and size of the plant (Fig. 5). Likewise, the analysis of the length of stem and roots separately reveals that there was no positive greater effect than Hoagland-treated plants on the size in any of the cases, except in the previously mentioned purge. It is highlighted that in all cases the results are positive since there is no significant difference in growth with respect to the synthetic control used or even it is greater in the case of the purge at 0.5 g·L^−1^.

In addition, the results show that the biomass concentrations higher than 0.5 g·L^−1^ can be harmful since a more concentrated biomass involves higher concentration of protein and, therefore, of amino acids or polyamines, which could be inhibiting plant growth, already reported by previous studies (e.g. Navarro-Lopez et al. 2020; Ferreira et al. 2021).

#### Microalgae biomass as biopesticide against *Fusarium oxysporum* and *Rhizoctonia solani* fungi

**Fig. 6:**
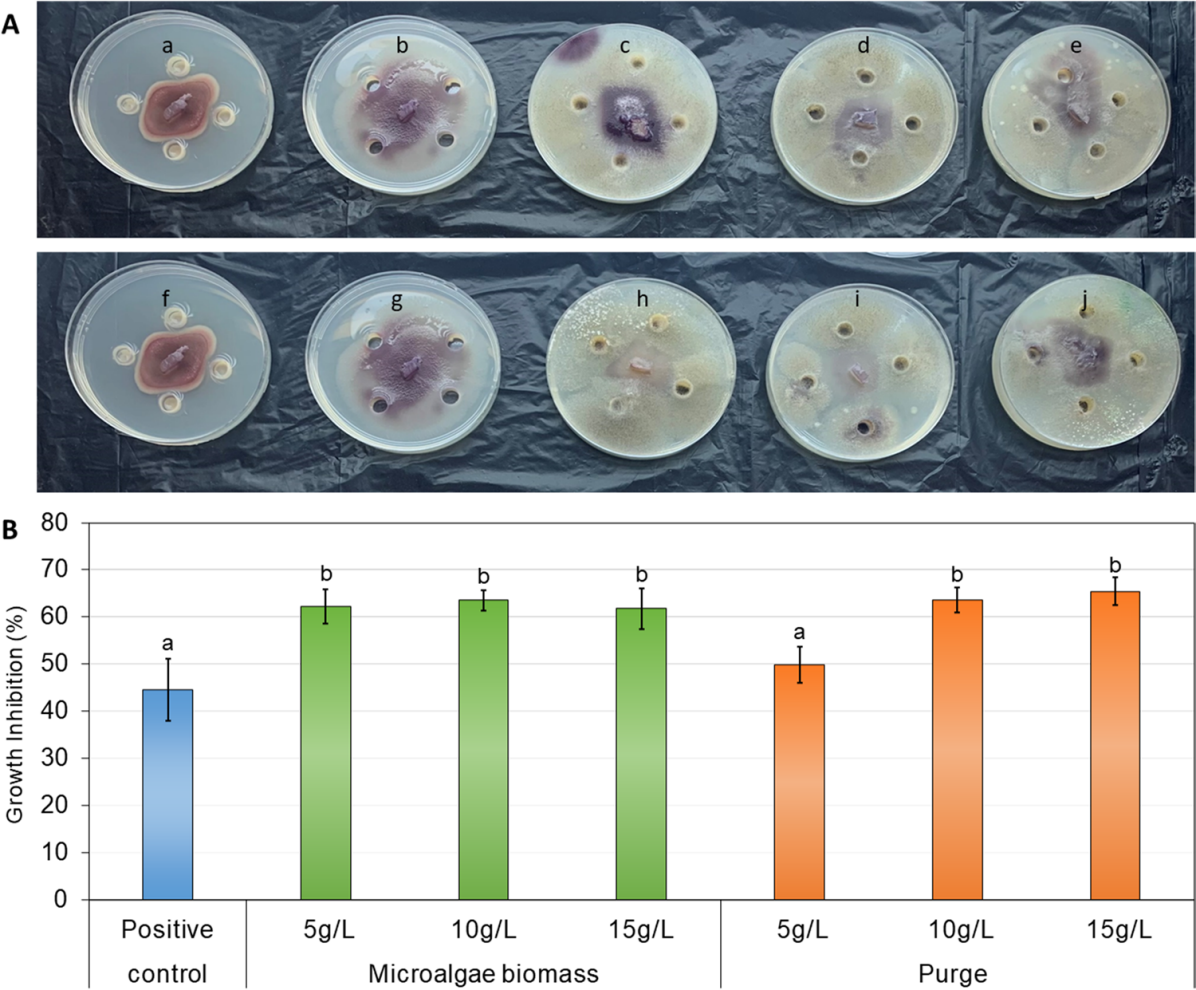
**A** Biopesticide activity for *Fusarium oxysporum* of the study suspensions: (a) and (f) Commercial biopesticide; (b) and (g) distilled water; microalgae biomass: (c) 5g·L^−1^; (d) 10 g·L^−1^; (e) 15 g·L^−1^; purge MPBR: (h) 5 g·L^−1^ (i) 10 g·L^−1^ (j) 15 g·L^−1^. The error bars indicate the standard deviation (*n* = 3). **B** Percentage of inhibition (*I*) of *Fusarium oxysporum* by harvested biomass in MPBR and MPBR purge both at different concentrations (5, 10, and 15 g·L^−1^), negative control (distilled water) and positive control (rovral 10 mg·L^−1^). The error bars indicate the standard deviation (*n* = 3)

Several microorganisms, including microalgae, have shown pesticide activity in previous studies (Asimakis et al. 2022; Braun and Colla 2022; Costa et al. 2019). In this study, some samples have been tested to determine their potential impact on the growth of the fungi *Fusarium oxysporum* or *Rhizoctonia solani* (Fig. 6). For this, different suspensions of the biomass harvested from the MPBR (microalgae-bacteria consortium) and purge at concentrations of 5, 10 and 15 g·L^−1^ were tested. In addition, negative and positive control consisting of distilled water and a commercial biopesticide respectively, were used.

In this case, the samples were not effective for the inhibition of the *R. solani*, but they were effective for the *F. oxysporum*. Figure 6 A is possible to observe the inhibition of the growth of these last fungus in all the samples used, compared to the negative control (distilled water).

The quantification of these reported inhibitions has been represented in Fig. 6B. In this case, the inhibition is not related to the biomass concentration, but related to the biomass characteristics. So, the microalgae-bacteria consortium, even at the lowest concentration of 5 g·L^−1^ allowed inhibition greater than 60%. While the purge inhibited by 49.9% at a concentration of 5 g·L^−1^ and exceeded 60% in the rest of the tested concentrations. In addition, it should be noted that the inhibition of the *F. oxysporum* using a commercial fertiliser was 44.6% in all the cases. Ferreira et al. (2021) showed inhibition percentages from different microalgae, being 5 g·L^−1^ the best concentration, which reached 49.5% of inhibition for *C. vulgaris* (same genus of the consortium of this study). Also, Ranglová et al. (2021) determined that *C. vulgaris* cultivated in wastewater showed a greater antibacterial and antifungal activity than those cultivated in BG-11, doubling its effectiveness, and reaching results of up to 32.0% effectiveness against *Fusarium oxysporum*.

Overall, as the lower concentrations have reported the best inhibition, it should be determined if lower concentrations could achieve a competitive percentage of inhibition. In addition, the biopesticide potential should be evaluated in other pathogenic microorganisms since Ranglová et al. (2021) indicates better efficiencies in *C. vulgaris* cultivated in wastewater compared to those cultivated in BG-11 against oomycetes such as *Phytophthora capsiciy* and *Pythium ultimum* and bacteria, such as *Xanthomonas campestris*, *Pseudomonas syringae* and *Pectobacterium carotovorum*.

## Conclusion

The proposed system in this study uses a combination of an UASB reactor and a membrane photobioreactor, allowing a potential biogas production of 0.25 kWh·m^−3^. Moreover, nutrients were assimilated by microalgae (2.0 to 5.9 mg N·L^−1^·d^−1^ and 4.8 to 6.0 mg P·L^−1^·d^−1^) which was successfully tested as biostimulant and fertiliser to maize plants, and pesticide against *Fusarium oxysporum*. Membrane permeate resulted in regenerated water according to European guidelines for water reuse for agricultural irrigation (European Commission 2020). Furthermore, membrane filterability was stable, preventing severe membrane fouling despite the colloidal nature of the suspensions. However, optimising the operating conditions of the MPBR and physical and chemical cleanings of the membrane module will allow a better performance.

To sum up, the combined system conceived as a parallel process integrated with the conventional systems will allow sustainable nutrients recovery, generating valuable biomass and an effluent suitable for reuse on sustainable agriculture.

### Supplementary Information

Below is the link to the electronic supplementary material.

Supplementary file1 (DOCX 2360 KB)

Supplementary file2 (DOCX 507 KB)

## Data Availability

The authors declare that the data supporting the findings of this study are available within the paper and its Supplementary Information files. Should any raw data files be needed in another format they are available from the corresponding author upon reasonable request. Source data are provided with this paper.
