# Correction to: Resources recovery from domestic wastewater by a combined process: anaerobic digestion and membrane photobioreactor

**DOI:** 10.1007/s11356-024-34619-6

**Published:** 2024-08-09

**Authors:** Elvira Ferrera, Ignacio Ruigómez, Carolina Vela‑Bastos, Alice Ferreira, Luisa Gouveia, Luisa Vera

**Affiliations:** 1 Departamento de Ingeniería Química y Tecnología, Farmacéutica, Facultad de Ciencias, Universidad de La, Avenida Astrofísico Francisco Sánchez S/N, 38206 LagunaLa Laguna, Spain; 2 LNEG ‑ UBB ‑ National Laboratory of Energy and Geology I.P., Bioenergy and Biorefineries Unit, Estrada Do Paço Do Lumiar 22, 1649‑038 Lisbon, Portugal; 3 GreenCoLab ‑ Green Ocean Technologies and Products, Collaborative Laboratory, CCMAR, Algarve University, Faro, Portugal

**Correction to: Environmental Science and Pollution Research**

10.1007/s11356-024-34468-3

Figures 2 and 3 are interchanged. The captions don't correspond with the figures.

Figure 2c doesn't exist.

The reference of the financial support of PID2021-125404OB-I00 project that appears in acknowledgement and funding is not a doi (https://doi.org/10.13039/501100011033/FEDER) it is a reference. The correct and complete financial support reference of this project is “MCIN/AEI/10.13039/50110001103/FEDER Una manera de hacer Europa”.

The Original article has been corrected.

