# Supplementary material for: Resources recovery from domestic wastewater by a combined process: anaerobic digestion and membrane photobioreactor
Source: Environ Sci Pollut Res Int. 2024 Jul 30;31(37):49560–73. doi: 10.1007/s11356-024-34468-3 (PMC11324692; doi:10.1007/s11356-024-34468-3)
Supplement: Supplementary file 2 — Supplementary file2 (DOCX 507 KB) [file 11356_2024_34468_MOESM2_ESM.docx]

**Supplementary Information (SI)**

**Resources recovery from domestic wastewater by a combined process: anaerobic digestion and membrane photobioreactor**

***Environmental Science and Pollution Research***

**Elvira Ferrera****^1^, Ignacio Ruigómez^1^, Carolina Vela-Bastos^2,3^, Alice Ferreira^2^, Luisa Gouveia^2,3^, Luisa Vera^1*^**

^1^ Departamento de Ingeniería Química y Tecnología Farmacéutica, Facultad de Ciencias, Universidad de La Laguna, Avenida Astrofísico Francisco Sánchez s/n, 38206 La Laguna, Spain. E-mail: [eferrera@ull.es](mailto:eferrera@ull.es) ; [isempere@ull.es](mailto:isempere@ull.es); luvera@ull.edu.es

^2^ LNEG - UBB - National Laboratory of Energy and Geology I.P., Bioenergy and Biorefineries Unit, Estrada do Paço do Lumiar 22, 1649-038 Lisbon, Portugal. E-mail: [alice.ferreira@lneg.pt](mailto:alice.ferreira@lneg.pt); [luisa.gouveia@lneg.pt](mailto:luisa.gouveia@lneg.pt)

^3^ GreenCoLab - Green Ocean Technologies and Products Collaborative Laboratory, CCMAR, Algarve University, Portugal. E-mail: [carolinabastos@greencolab.com](mailto:carolinabastos@greencolab.com)

*Corresponding author. E-mail address: [luvera@ull.edu.es](mailto:luvera@ull.edu.es)


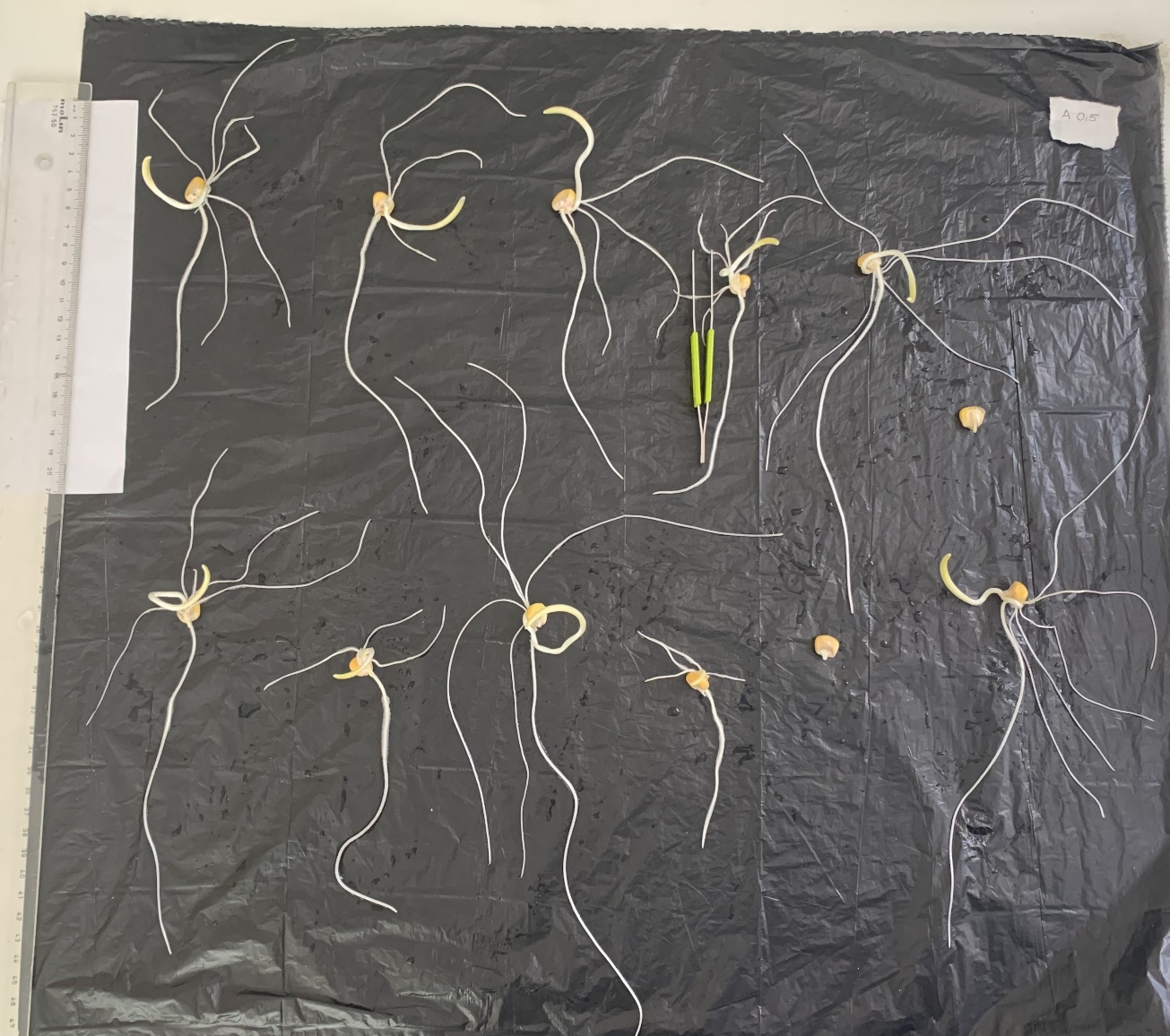


**Figure S2.** Germinated seeds.
